# Development of a method for phycocyanin recovery from filamentous cyanobacteria and evaluation of its stability and antioxidant capacity

**DOI:** 10.1186/s12896-021-00692-9

**Published:** 2021-06-16

**Authors:** Jinichi Aoki, Daisaku Sasaki, Munehiko Asayama

**Affiliations:** 1 College of Agriculture, Ibaraki University, 3-21-1 Ami, Ibaraki 300-0393, Japan; 2 United Graduate School of Agricultural Science, Tokyo University of Agriculture and Technology, 3-5-8 Saiwai-cho Fuchu-shi, Tokyo 183-8509, Japan; 3 BioX Chemical Industries Co. Ltd., 2-20-11 Inokuchidai, Nishi-ku, Hiroshima 733-0844, Japan

**Keywords:** Antioxidant, *Arthrospira platensis*, Cell lysis, *Limnothrix* sp., Phycocyanin, *Pseudanabaena* sp.

## Abstract

**Background:**

Most commercial phycocyanins are extracted from a filamentous cyanobacterium, *Arthrospira* (*Spirulina*) *platensis.* Owing to the expenses of culture and complexities of the physical and chemical methods of phycocyanin purification, a more effective and simple method is required.

**Results:**

We developed a new method for efficiently recovering the blue pigment protein, phycocyanin, from unique filamentous cyanobacteria, *Pseudanabaena* sp. ABRG5-3 and *Limnothrix* sp. SK1-2-1. The cells were cultivated in economy medium BG11 and lysed by adding water in a 1:16 ratio of wet cells to water. After extraction and purification, 28–30% dry cell weight of phycocyanin was obtained and its purity was confirmed. The stabilities of the phycocyanins at different pH in the presence of high temperature and light conditions and their antioxidant abilities were assessed. Results indicated that the phycocyanins were stable and possessed antioxidant properties. Interestingly, the *Pseudanabaena* phycocyanin was less likely to deteriorate under acidic conditions.

**Conclusions:**

Overall, we developed a promising and novel method for producing high functional phycocyanin concentrations at a low cost. The possibilities of adapting this new phycocyanin biorefinery to unique bioreactor utilization have also been discussed.

**Supplementary Information:**

The online version contains supplementary material available at 10.1186/s12896-021-00692-9.

## Background

Currently, the interest in producing useful substances such as lipids, sugars, proteins, and pigments from microalgae has increased because of the latter’s ability to reuse carbon dioxide, the cause of global warming. Photosynthetic organisms can effectively produce organic substances from inorganic substances, which have been utilized in microalgal biorefineries [1, 2]. In addition to the high biomass productivity, the generation of useful materials is another important feature of biorefineries [3, 4].

Phycocyanin, a photosynthetic blue pigment protein found in cyanobacteria (blue green algae), grey algae, and red algae, is a heterodimer of α (CpcA, C-phycocyanin alpha-subunit gene product) and β (CpcB, C-phycocyanin beta-subunit gene product) subunits of the phycobiliprotein monomer, which associates with phycocyanobilin as its chromophore [5] via a thioether bond [6] (Additional file 1: Figure S1). Phycocyanin has been used as a nutritional component [7], natural dye [8], fluorescent marker [9, 10], antioxidant [11], and food colouring agent [12, 13], and in cosmetics such as lipstick and eyeliner [14]. As phycocyanin is soluble in water, cells have to be first disrupted using physical or chemical methods for extracting the pigment. The physical methods’ expenses and complexities, such as repeated freeze-thawing of cyanobacterial cells [15] or ultrasonication [16] are high. The expenses associated with the chemical methods are also high, as phosphate buffer, acetate buffer, hexane, and solvents with sodium chloride or sodium azide are used [15, 17]. Most commercial phycocyanins are extracted from a filamentous cyanobacterium, *Arthrospira* (*Spirulina*) *platensis.* Owing to the expenses and complexities of the physical and chemical methods of phycocyanin purification, a more effective and simple method is required.

This study aimed to develop a new method for effectively extracting and purifying phycocyanin from other useful cyanobacteria. Toward this, we used two unique filamentous cyanobacteria, *Pseudanabaena* sp. ABRG5-3 [18–20] and *Limnothrix* sp. SK1-2-1 [21] for extracting phycocyanin, as they have previously been used in the dual (solid and liquid)-phase cultivation system (DuPHA) for production of biofuels [22]. The ABRG5-3 strain exhibits a unique feature of auto cell-lysis and can produce C17 alkane (heptadecane, C_17_H_36_) as biofuel [23, 24]. The SK1-2-1 strain also exhibits a unique feature of flocculation with extracellular polysaccharide accumulation and can produce C15 alkane (pentadecane, C_15_H_32_) as biofuel [21]. We found the condition of filamentous cyanobacteria lysis. Furthermore, the stabilities and antioxidant properties of the purified phycocyanins were compared to those of a commercial phycocyanin.

## Results

### Media and phycocyanin production

**Fig. 1:**
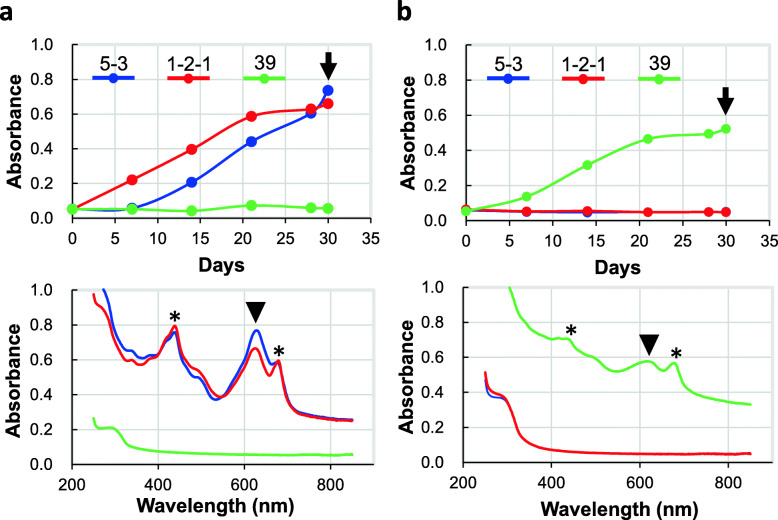
Media and phycocyanin production. **a** Cells were cultivated in BG11 medium and cell turbidities were measured at 618 nm (top). The sample was harvested after 30 days (arrow) and the absorption spectrum was measured from 250 nm to 830 nm (bottom). The positions of the maximum absorbance at 618 nm for phycocyanin, and at 435 nm and 680 nm for chlorophyll *a,* are shown with an arrowhead and asterisks, respectively. 5-3, ABRG5-3; 1-2-1, SK1-2-1; 39, NIES-39. **b** Cells were also cultured in SOT medium (top). The wave scan was performed using the same procedure as shown in (**a**) (bottom)

*Pseudanabaena* sp. ABRG5-3, *Limnothrix* sp. SK1-2-1, or *Arthrospira* (*Sprulina*) *platensis* can be cultivated on BG11 or SOT medium, respectively [15, 18, 21, 22]. The maximum absorbance at 618 nm was sequentially measured to investigate phycocyanin production until 30 days and the results are shown in Fig. 1 (upper panels). The relation between the absorbance value at 618 nm and the amount of phycocyanin is proportional. Significant amounts of phycocyanin accumulated in ABRG5-3 and SK1-2-1, but not in NIES-39 in the BG11-medium (panel a), when the filamentous cyanobacteria were cultivated in the BG11 (panel a) or SOT (panel b) medium for 30 days. In contrast, NIES-39 grew well and produced phycocyanin in the SOT medium, although ABRG5-3 and SK1-2-1 did not grow in SOT medium (panel b). Thus, we confirmed that abundant phycocyanins accumulated by day 30 in the respective cases (Fig. 1, lower panels). In particular, the peak values at 618 nm, indicating phycocyanin production, were more significant in ABRG5-3/SK1-2-1 than in NIES-39, compared to the values observed at 680 nm for chlorophyll *a*. Thus, BG11 or SOT medium is appropriate for phycocyanin production using ABRG5-3/SK1-2-1 or NIES-39, respectively.

### Lysis of cyanobacteria via water addition

**Fig. 2:**
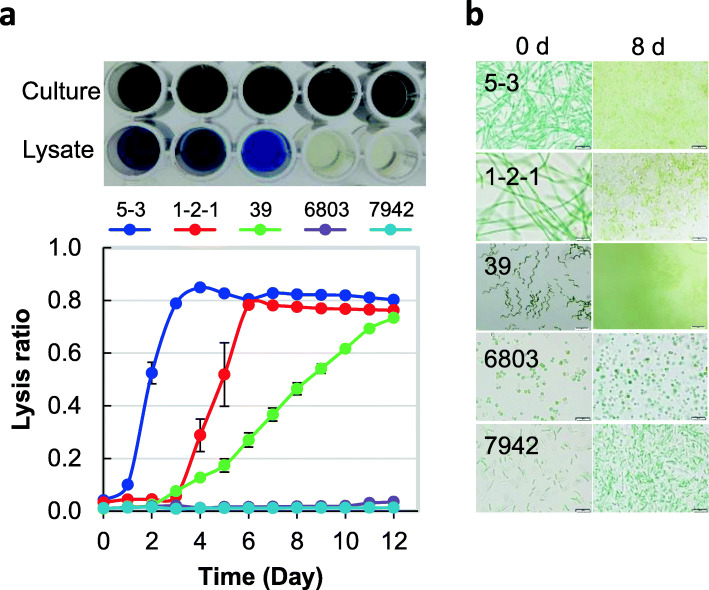
Cell lysis via addition of water. **a** Water (30 mL) was added to the cell pellet corresponding to 1 L of the culture, and the mixture was stirred and allowed to stand in the dark. Each cyanobacterial supernatant and culture were placed in a 96-well plate and incubated for 12 days in the dark as shown at the top. Absorbance (OD618) of the solution and supernatant were measured daily under these conditions for determining phycocyanin content. Lysis ratio is shown at the bottom. The mean of the values obtained from three independent experiments is shown together with standard errors. **b** Live cells (0 day) and lysed or live cells (8 days) under the static/dark conditions were observed using an optical microscope

Until now, various methods for cell disruption and phycocyanin extraction from cyanobacterial cells have been examined (Additional file 2: Table S1). This study showed a new method for easy lysis and efficient extraction using ABRG5-3/SK1-2-1/NIES-39 (filamentous), *Synechocystis* sp. PCC 6803 (unicellular), and *Synechococcus elongatus* PCC 7942 (bacillary). The lysis ratio was evaluated as the ratios of OD618 of lysate to OD618 of culture, and the results are shown in Fig. 2a. Although cocci PCC 6803 and bacillary PCC 7942 did not lyse, the number of days required for the lysis of filamentous cyanobacteria ABRG5-3, SK1-2-1, and NIES-39 varied, although almost 80% lyses occurred within 2–12 days. We also confirmed that the cells lysed on the 8th day in the filamentous cyanobacteria, but not in other cyanobacteria (Fig. 2b). These results indicated that the new method was efficient for lysis of filamentous cyanobacteria.

**Fig. 3:**
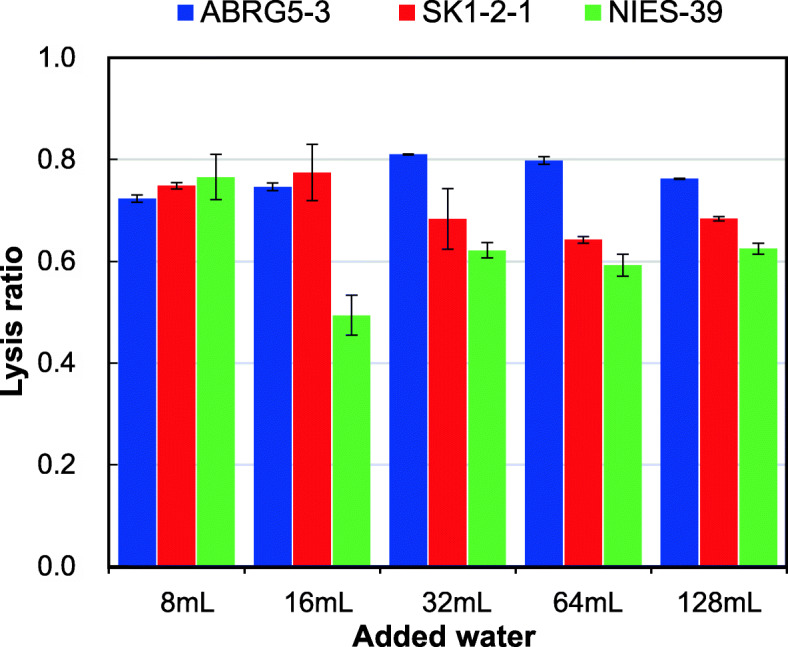
Cell lysis by adding varying amounts of water. Water (8 mL, 16 mL, 32 mL, 64 mL, and 128 mL) was added to each wet cell (1 g) of ABRG5-3, SK1-2-1, or NIES-39 culture, and the lysis ratio in the dark was calculated on the sixth day. The mean of the values obtained from three independent experiments is shown together with standard errors

Next, we investigated the ratio of the added water volume to the wet cell pellets, as shown in Fig. 3. The results indicated that effective lysis occurred at various rates for volume ratios 1:8, 1:16, 1:32, 1:64, and 1:128 for ABRG5-3/SK1-2-1 on the 6th day after addition of water. We used the 1:16 volume ratio in subsequent experiments, as high viscosity and small amount of PC volume might be problematic for phycocyanin purification. Similar tendency was observed for ABRG5-3 or SK1-2-1 from the 2nd to the 12th day (data not shown).

### Phycocyanin purification using activated carbon

**Fig. 4:**
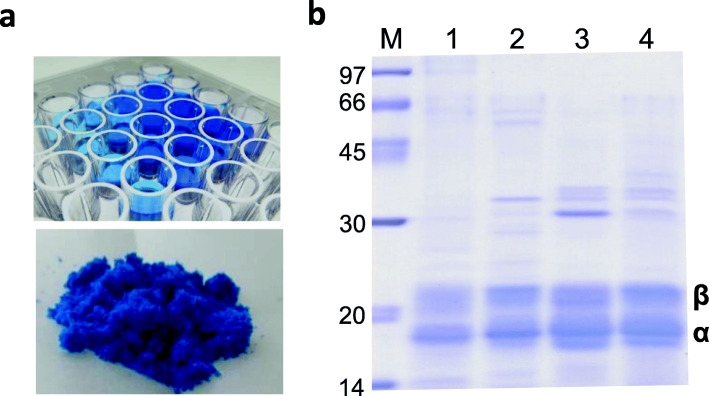
Phycocyanin purification. **a** The extracted PC in 96 wells and PC powder obtained after a freeze-drying from the PC solution are shown. **b** SDS-PAGE of purified phycocyanin; purity was also reconfirmed by the presence of single bands of the α-subunit (18.4 kDa) and β-subunit (21.3 kDa) on SDS polyacrylamide gel. M: Low-molecular-weight marker, 1: *Pseudanabaena* sp. ABRG5-3, 2: *Limnothrix* sp. SK1-2-1, 3: *Spirulina platensis* NIES-39, 4: Commercial product (phycocyanin from *Spirulina*)

Phycocyanin was purified from the respective cyanobacterial lysates using activated carbon as described in Methods (Additional file 1: Figure S2). Using this method, 0.12 g (30% in dry cell weight), 0.13 g (29% in dry cell weight), and 0.16 g (21% in dry cell weight) phycocyanin were extracted from 1 L culture of ABRG5-3, SK-1-2-1, and NIES-39, respectively. The purified aqueous solution (upper panel) and powder post-freeze-drying (lower panel) of ABRG5-3 phycocyanin are shown in Fig. 4a. The purity of the fraction was verified using SDS-PAGE (Fig. 4b), which showed that compared to a commercial purified product from *Spirulina* (lane 4), the α- (18.4 kDa) and β- (21.3 kDa) subunits of phycocyanin were the main bands on the gel from ABRG5-3 (lane 1), SK1-2-1 (lane 2), and NIES-39 (lane 3). Furthermore, analysis of the absorption spectrum of phycocyanin from ABRG5-3 and SK1-2-1 indicated that miscellaneous proteins and/or nucleic acids can be removed from the phycocyanin solution (Additional file 1: Figure S3). We also confirmed the purity from the ratio of the absorbances at 618 and 280 nm, which were 3.10, 2.14, or 1.76 for phycocyanin from ABRG5-3, SK1-2-1, or NIES-39, respectively (Additional file 2: Table S1).

### Stability of phycocyanin depended on pH in the presence of heat and light

**Fig. 5:**
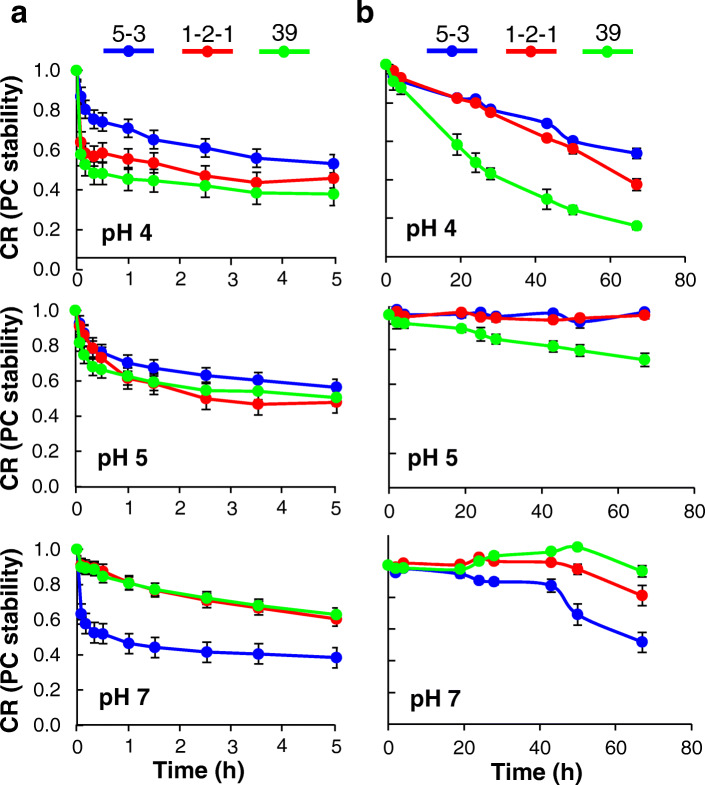
Stability of phycocyanin depended on pH in the presence of heat and light. **a** The pH of PC from ABRG5-3, SK1-2-1, and NIES-39 was varied (phosphate-citrate buffer) at 55 °C. The relative phycocyanin concentration (CR) indicating PC stability were measured. The mean of the values obtained from three independent experiments is shown together with standard errors. **b** CR values were also measured at different pH in which phycocyanin was exposed to high total light intensity (100 μmol photons m^− 2^ s^− 1^) at 30 °C for the indicated durations

As phycocyanin is a blue pigment protein, it undergoes heat denaturation. In this study, the heat resistance of phycocyanin extracted from ABRG5-3, SK1-2-1, and NIES-39 was investigated in a phosphate-citrate buffer at pH 4, 5, and 7 under various temperature. Results indicated that the heat denaturation as fading of phycocyanin colour significantly occurred above 50 °C and approximate 50–60% stabilities of phycocyanin were observed at 55 °C (Additional file 1: Figure S4). Thus, we more tested the stability at 55 °C under various incubation times. Results showed that phycocyanin from ABRG5-3 was more stable than those from SK1-2-1 and NIES-39 at pH 4 and 5. However, the phycocyanin from ABRG5-3 was less stable than those from SK1-2-1 and NIES-39 at pH 7 (Fig. 5a). Next, photodegradation of phycocyanin was investigated in phosphate-citrate buffer at pH 4, 5, and 7 after continuous irradiation with 100 μmol photons m^− 2^ s^− 1^ at 30 °C. Results showed that the ABRG5-3 phycocyanin was less likely to deteriorate under acidic conditions (Fig. 5b).

### Antioxidant capacity of phycocyanin

**Fig. 6:**
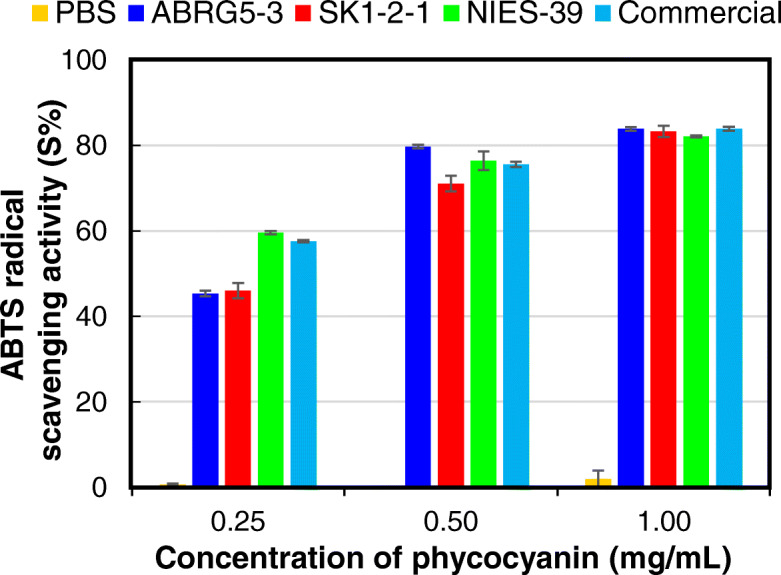
Antioxidant activity of the purified phycocyanin. ABTS assay was performed and radical scavenging activities (S%) are shown. The corresponding phycocyanin concentrations in PBS (phosphate-buffered saline) are shown. ABRG5-3 (blue), SK1-2-1 (red), NIES-39 (green), commercial *Spirulina* sample (light blue), or PBS only (yellow). The mean of the values obtained from three independent experiments is shown together with standard errors

Phycocyanins are known to possess antioxidant properties and are important as food additives. The antioxidant activities of different concentrations of phycocyanin from ABRG5-3, SK1-2-1, and NIES-39 were estimated using the ABTS method described in the “Determination of the antioxidant potential of phycocyanin” subsection of the Methods. The results indicated that no activity was observed when PBS was used as the negative control. In contrast, significant antioxidant activities of ABRG5-3 and SK1-2-1 were confirmed when commercial *Spirulina* samples or those from NIES-39 were used as positive controls in the concentration ranging from 0.25 to 1.00 mg mL^− 1^ (Fig. 6).

## Discussion

### Media and phycocyanin production

The SOT medium contains abundant NaHCO_3_, resulting in highly alkaline conditions with HCO_3_^−^, and is approximately thrice as expensive as BG11 (data not shown). Thus, cultivation using BG11 medium might be advantageous for abundant phycocyanin accumulation in ABRG5-3 and SK1-2-1, as is evident from the ratio of maximum absorbance of phycocyanin (at 618 nm) versus that of Chl *a* (at 435 and 680 nm), compared to that for NIES-39 cultivated in SOT medium (Fig. 1, bottom panels). Light photons were measured using a LI-COR instrument (Lincoln, NE, USA) and the range from 20 to 30 μmol photons m^− 2^ s^− 1^ was determined appropriate for cyanobacterial growth under indoor conditions [21, 22]. Light intensity could be applicable not only in this study but also to large-scale economical indoor/outdoor cultivations in the future. We confirmed that strains could grow under the light intensity and the growth curves are presented in Fig. 1. Nitrogen concentration was not measured in this study. However, C-phycocyanin apparently accumulated and did not decrease during 30 days which is linked to the cell growth, and indicates that nitrogen sources still remained in the culture (Fig. 1). We previously reported that poor cell growth and auto cell-lysis occurred when nitrogen-depleted medium was used for ABRG5-3 cultivation [18]. This also showed that nitrogen sources could not be completely consumed in the medium.

### Lysis of cyanobacteria via water addition

Until now, phycocyanin has been mainly extracted from the cyanobacteria *Spirulina* using phosphate buffer (Additional file 2: Table S1). Thus, this is the first study to extract phycocyanin via the addition of only water and incubation in the dark (Additional file 1: Figure S1). Although the mechanism underlying the water-mediated cell lysis remains unknown, the vulnerability of filamentous cell structures or thylakoid-membrane structures under the darkness condition [18] may be due to water-mediated cell lysis. Of note, the 6 L-tank 30-day cell culture after inoculation did not become contaminated at any time point. Extraction of phycocyanin by water addition might be certainly a time-consuming process compared to other reported extraction methods and solvents. Nevertheless, due to its low cost this could be considered an attractive method. Careful handling is required to avoid contamination when phycocyanin extracts are utilized for edible or medical materials, but phycocyanin products extracted with deionized or tap water could be used for other materials (colouring and tracer etc.)

### Phycocyanin purification using activated carbon

Phycocyanin has been previously recovered using column chromatography, ammonium sulfate purification, two-layer separation with phosphate buffer/hexane, and dialysis [15–17, 25]. However, these methods may sometimes be cumbersome and expensive. In contrast, the method using activated carbon is comparatively simple and inexpensive, leading to recovery of highly pure phycocyanin from filamentous cyanobacteria ABRG5-3 and SK1-2-1, and *Spirulina*. The yield is known to range from 7.2 to 10.2% from dry cell weight of *Spirulina* (Addtional file 2: Table S1). In contrast, 2.1- to 4.2-fold higher yields were obtained using activated charcoal (Additional file 2: Table S1). Purity ranging from 1.8 to 3.1 by activated carbon indicated significantly more efficacy than those of other cases [15–17, 25], while the range from 0.75 to 0.91 is available for food additive even if it is lower than that of 4.6 as analytical grade obtained from the column chromatography. The PC recovery-yields were 30.4% from *Pseudanabaena* sp. ABRG5-3, 28.9% from *Limnothrix* sp. SK1-2-1, and 21.1% from *Arthrospira* NIES-39 (Addtional file 2: Table S1), despite being from filamentous cyanobacteria. Their distinct yields might depend on cell (membrane) structures and/or extracellular polysaccharides produced (EPS) from the cells. For example, it has been reported that auto cell-lysis of ABRG5-3 depends on soft cell structure [18, 19] and this might accelerate cell lysis by the water addition. On the other hand, *Arthrospira* produce abundant EPS on cell surface which might reduce the efficiency of the PC extraction [26]. Anyway, this new method could be conducted with optional steps for obtaining high purity without contaminations, such as the use of ultraviolet sterilization, filter and/or column chromatography, depending on utilization purposes.

### Stability of phycocyanin depended on pH in the presence of heat and light

In ABRG5-3 phycocyanin, the amino acid sequences around the cysteine required for binding to phycocyanobilin (pigment) in CpcA and CpcB are not particularly rich in acidic amino acids (Additional file 1: Figure S5). This may indicate that the entire protein structure, and not the local amino acid sequence, affects the stability at each pH condition.

### Phycocyanin biorefinery

Although the stabilities and antioxidant properties of the phycocyanins from unique cyanobacteria were confirmed as food additives in this study, phycocyanins have also been reported to act as fluorescent markers [9, 10], cosmetics [14], and anticancer agents [27]. Hence, a wide range of phycocyanins can be used for developing new phycocyanin biorefineries, which can be utilized for developing bioreactors of natural or engineered microalga [8]. For example, effective biomass can be produced using a unique bioreactor, DuPHA [22], which involves dual solid and liquid phases under autotrophic, mixotrophic, or heterotrophic culture conditions. We would apply this developmentary method not only for ARBG5-3, SK1-2-1, and NIES-39 but also for all filamentous cyanobacteria.

## Conclusions

This is the first study reporting phycocyanin extraction from unique filamentous cyanobacteria, *Pseudanabaena* sp. ABRG5-3 and *Limnothrix* sp. SK1-2-1, using a water-adding procedure. Phycocyanin yield was approximately 30% of the dry cell weight using a low-cost cultivation medium and extraction/purification steps. The purified phycocyanins showed considerable thermal (55 °C) and photo (100 μmol photons m^− 2^ s^− 1^) stability depending on the pH. In addition, they possessed potential antioxidant activities that can be used as a food additive etc.

## Methods

### Bacterial strains

The filamentous cyanobacteria *Pseudanabaena* sp. ABRG5-3 [18–20] and *Limnothrix* sp. SK1-2-1 [21] have been isolated and published. The filamentous cyanobacterium *Arthrospira platensis* NIES-39 was obtained from the National Institute for Environmental Studies (NIES: Tsukuba, Japan). Cyanobacteria *Nostoc* (*Anabaena*) sp. PCC 7120, *Synechococcus elongatus* PCC 7942, and *Synechocystis* sp. PCC 6803 were obtained from the Pasteur Institute (Paris, France).

### Cultivation conditions

All cyanobacteria, except for NIES-39, were grown in sterilized BG11 liquid medium [28] under LED white-light (RGB mix, approximately 20 μmol photons m^− 2^ s^− 1^) irradiation at 30 °C with shaking (100 rpm) in Erlenmeyer flasks (50 mL medium in 100 mL flask) for 3 weeks. This culture was transferred to a 6 L BG11 sterile liquid medium in a 6 L tank with aeration (3 L air min^− 1^) and cultivated under the LED irradiation-condition at 30 °C for a month. The strain NIES-39 was grown in sterilized SOT liquid medium [29] under the same cultivation condition.

### Measurement of absorption spectrum and dry cell weight

A 200 μL aliquot of the cell culture was collected and placed in a well of a 96-well microtiter plate. The absorption spectrum was measured from 250 to 830 nm using a Multiskan Go spectrometer (Thermo Fisher Co. Ltd., Tokyo, Japan). The cyanobacterial cells cultured in the 6 L tank were harvested via centrifugation and freeze-dried using a vacuum freeze dryer (FZ-Compact; Labconco Co. Ltd., Kansas City, USA). The dry cell weight was measured by using electric balance (AY303; Sartorius Co.Ltd., Göttingen, Germany).

### Phycocyanin extraction via water addition

#### Lysis ratio

The lysis ratio was determined as follows:

$$ \mathrm{Lysis}\kern0.5em \mathrm{rate}=\frac{\mathrm{OD}618\kern0.5em \mathrm{of}\kern0.5em \mathrm{lysate}}{\mathrm{OD}618\kern0.5em \mathrm{of}\kern0.5em \mathrm{culture}} $$

#### Wet cell lysis from 1 L culture

The cells and the culture solution were separated via centrifugation (4300×*g*, 30 min), following which, 30 mL deionized water DW was added to cyanobacterial cells corresponding to 1 L of the culture solution in a 50 mL centrifuge tube, stirred, and allowed to stand in the dark. In this state, 1 mL of the concentrated solution was removed daily, 200 μL transferred to a 96-well plate, and the absorbance at 618 nm was measured. At the same time, 0.8 mL of the concentrated culture sample was centrifuged (3100×*g*, 5 min), followed by transfer of 200 μL supernatant to a 96-well plate and measurement of the absorbance at 618 nm. After the measurement, the 1 mL sample was returned to its original position, 30 mL, and allowed to stand in the dark. When the lysis ratio was approximately 0.8, the concentrated culture solution was centrifuged in a 50 mL tube at 4300×*g* for 30 min and the supernatant containing phycocyanin was collected.

#### Differences in lysis rate with the amount of added water

Next, to compare the lysis rate differences with the amount of added water, the collected ABRG5-3, SK1-2-1, and NIES-39 culture liquids were separated into bacterial cells and culture solution via centrifugation. To 1 g wet weight of bacterial cells, 8 mL, 16 mL, 32 mL, 64 mL, and 128 mL DW were added, and the mixture was stirred and placed in the dark at 30 °C. The ABRG5-3, SK1-2-1, and NIES-39 cells were lysed and phycocyanin was eluted in water within a few days. The lysis rate was calculated using the above formula after every 2 days.

### Purification and purity assessment of phycocyanin

The aqueous supernatant containing phycocyanin described in the former section was mixed with 0.5% (w/v) activated carbon and stirred at room temperature for 15 min. Subsequently, the purified phycocyanin aqueous fraction was obtained via centrifugation at 4300×*g* for 30 min (10 °C). The phycocyanin purity was determined using the following formula [30] in which OD280 indicates the absorbance of total proteins and OD618 indicates the absorbance of phycocyanin.

$$ \mathrm{Phycocyanin}\ \mathrm{purity}=\frac{\mathrm{OD}618}{\mathrm{OD}280} $$

The aqueous solution of phycocyanin in a 50 mL centrifuge tube was freeze-dried using a vacuum freeze-dryer (FZ-Compact; Labconco Co. Ltd.), and the resulting phycocyanin powder was weighed directly. The purity and molecular weight of phycocyanin were further assessed using sodium dodecyl sulfate-polyacrylamide gel electrophoresis (SDS-PAGE) [31] as follows. SDS-PAGE loading buffer and the aqueous solution of phycocyanin (4 mg mL^− 1^) were mixed and heated at 95 °C for 2 min, following which 25 μL of the resultants were subjected to electrophoresis on a 12.5% SDS-polyacrylamide gel. After electrophoresis, the gel was stained with Coomassie Brilliant Blue R250 (Nacalai Tesque, Tokyo, Japan).

### Determination of phycocyanin stability

In this study, phycocyanin indicates C-phycocyanin, whereas it is denoted as C-phycocyanin (CPC) in CR (Relative concentration as PC stability) to distinguish it from allophycocyanin (APC). The stabilities of the phycocyanins at different pH in the presence of high heat and light conditions were assessed as follows. The respective phycocyanin powders (4 mg) were dissolved in 1 mL DW, then a 0.2 mL aliquot was mixed with 0.8 mL phosphate citrate buffer (0.15 M at pH 4, 5, or 7), resulting in 1 mL of the reaction solutions of ABRG5-3, SK1-2-1 and NIES-39 strains. The solutions were heated in a 55 °C hot water bath for 0, 0.08, 0.16, 0.33, 0.5, 1, 1.5, 2.5, 3.5, and 5 h, following which the absorbance at 618 nm and 652 nm were measured for determining the accumulation of CPC and APC, respectively (Fig. 5a). A lamp that emits 100 μmol photons m^− 2^ s^− 1^ (total intensity) was used to excite the phycocyanin solutions of pH of 4, 5, and 7 at 30 °C for 0, 2, 4, 19, 24, 28, 43, 50, and 67 h (Fig. 5b). The stability of phycocyanin (CR) was calculated by the ratio of summing the CPC and APC concentrations [32].

$$ \mathrm{CR}\;\left(\mathrm{PC}\;\mathrm{stability}\right)=\frac{\left(\mathrm{CPC}+\mathrm{APC}\right)\kern0.19em \mathrm{at}\kern0.19em \mathrm{specified}\kern0.17em \mathrm{time}}{\left(\mathrm{CPC}+\mathrm{APC}\right)\kern0.3em \mathrm{at}\kern0.3em 0\kern0.1em \min } $$

$$ \mathrm{CPC}\ \left(\mathrm{mg}\ {\mathrm{mL}}^{\hbox{-} 1}\right)=\frac{\mathrm{OD}618-0.474\times \mathrm{OD}652}{5.34} $$

$$ \mathrm{APC}\ \left(\mathrm{mg}\ {\mathrm{mL}}^{\hbox{-} 1}\right)=\frac{\mathrm{OD}652-0.208\times \mathrm{OD}618}{5.09} $$

### Determination of the antioxidant potential of phycocyanin

An ABTS [2,2′-azino-bis (3-ethylbenzothiazoline-6-sulfonic acid)] stock solution was prepared by mixing equal amounts of ABTS (7 mM) and potassium persulfate (2.45 mM). After storage in the dark for 18 h, the stock solution was diluted with phosphate buffered saline (PBS: 20 mM, pH 7.4) to make a working solution with an absorbance of 0.70 ± 0.05 at 734 nm. To measure the antioxidants potential, 20 μL aliquots of different concentrations of the phycocyanin solution diluted in PBS were mixed with 180 μL of ABTS working solution, and the absorbance (A) of the mixture was measured. PBS was used as the negative control. The absorbance at 734 nm was measured after incubation for 6 min at room temperature in the dark. The ABTS radical scavenging sample activity (S%, antioxidant ability) was determined using the following equation, where A_control_ is the absorbance of a blank control (a mixture of ABTS working solution and PBS), A_test_ is the absorbance of ABTS operating on the sample solution, and A_sample_ is the absorbance of the sample solution and PBS mixture [33].

$$ \mathrm{S}\%=\frac{{\mathrm{A}}_{\mathrm{control}}-{\mathrm{A}}_{\mathrm{test}}-{\mathrm{A}}_{\mathrm{sample}}}{{\mathrm{A}}_{\mathrm{control}}}\times 100 $$

### Microscopic observation

The cyanobacteria were observed under an optical microscope BX53/DP72 (Olympus, Tokyo, Japan) (× 1000 or 100) before and after lysis [18].

## Supplementary Information

**Additional file 1: Figure S1.** Phycocyanin in cyanobacteria. **Figure S2.** Phycocyanin extraction from cyanobacteria. **Figure S3.** Absorption spectrum for the phycocyanin fraction. **Figure S4.** Stability of phycocyanin depends on pH under heat. **Figure S5.** Alignment of the amino acids sequence of phycocyanin subunits.

**Additional file 2: Table S1**. Extraction, yield, and purity of phycocyanin from cyanobacteria.

## Data Availability

The datasets used and analyzed for the current study are available from the corresponding author upon reasonable request.
