# Supplementary material for: Development of a method for phycocyanin recovery from filamentous cyanobacteria and evaluation of its stability and antioxidant capacity
Source: BMC Biotechnol. 2021 Jun 16;21:40. doi: 10.1186/s12896-021-00692-9 (PMC8210370; doi:10.1186/s12896-021-00692-9)
Supplement: Supplementary file 1 — Additional file 1: Figure S1. Phycocyanin in cyanobacteria. Figure S2. Phycocyanin extraction from cyanobacteria. Figure S3. Absorption spectrum for the phycocyanin fraction. Figure S4. Stability of phycocyanin depends on pH under heat. Figure S5. Alignment of the amino acids sequence of phycocyanin subunits. [file 12896_2021_692_MOESM1_ESM.pdf]

Additional file 1: Additional figures

BMC Biotechnology

Development of a method for phycocyanin recovery from filamentous cyanobacteria and evaluation of its stability and antioxidant capacity

Jinichi Aoki[1,2], Daisaku Sasaki[3], Munehiko Asayama[1,2*]

[*]Correspondence: munehiko.asayama.777@vc.ibaraki.ac.jp

[1]College of Agriculture, Ibaraki University, 3-21-1 Ami, Ibaraki 300-0393, Japan
[2]United Graduate School of Agricultural Science, Tokyo University of Agriculture and Technology, 3-5-8 Saiwai-cho Fuchu-shi, Tokyo 183-8509, Japan
[3]BioX Chemical Industries Co. Ltd., 2-20-11 Inokuchidai, Nishi-ku, Hiroshima 733-0844, Japan

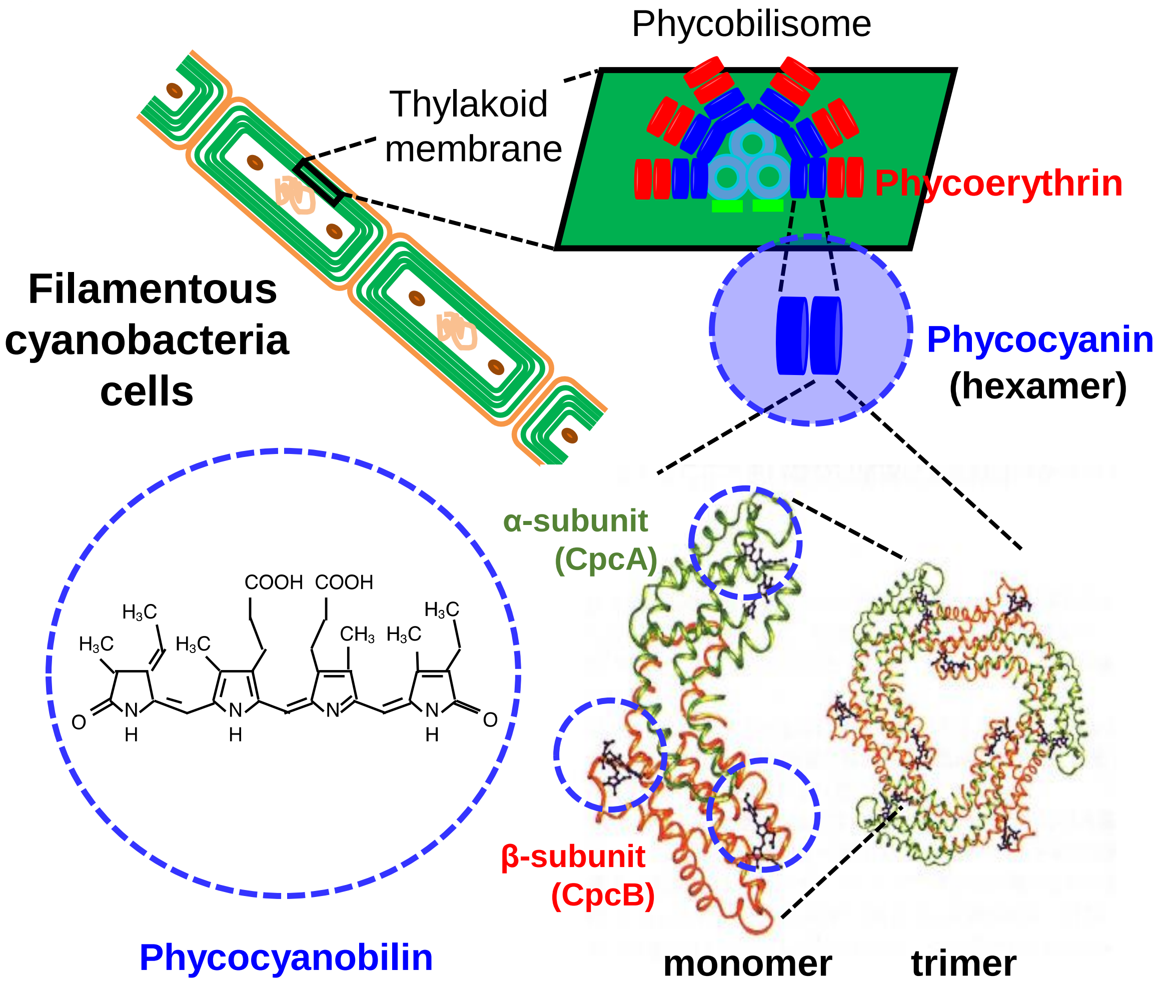

Figure S1 Phycocyanin in cyanobacteria.

Thylakoid membranes are in filamentous cyanobacteria cells. Phycobilisome is present on the thylakoid membrane. Phycobilisome consists of allophycocyanin, phycocyanin, phycoerythrin, and anchor protein. The structure of phycocyanin begins with assembly of phycobiliprotein monomers, which are heterodimers of α- and β-subunits. The structures of phycocyanin monomer and trimer crystals and phycocyanobilin are also shown. Phycocyanobilin is the chromophore binding to phycobiliprotein, as shown by the broken circles.

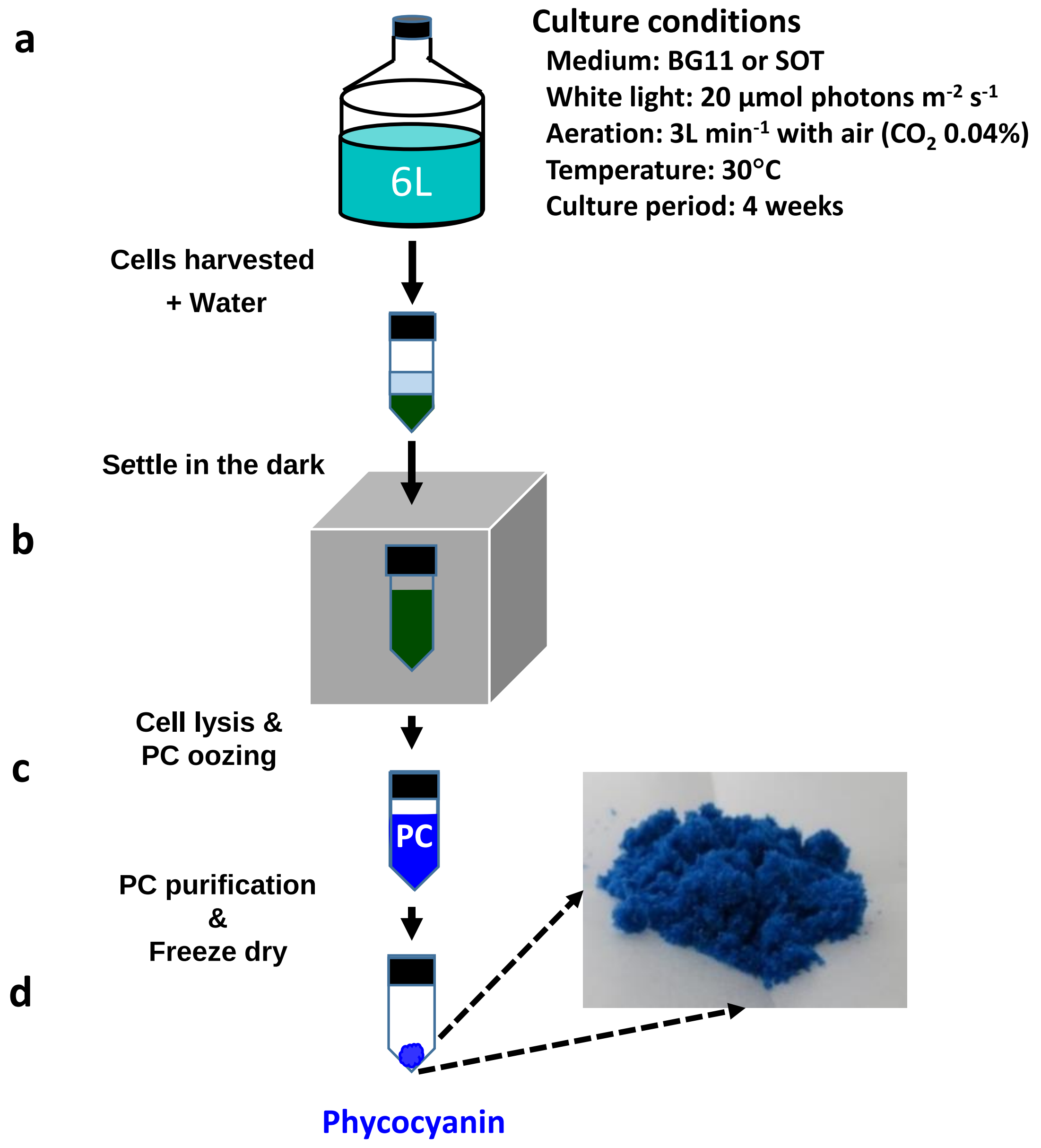

Figure S2 Phycocyanin extraction from cyanobacteria.

(**a**) Cyanobacteria were cultivated under the conditions shown in the panel. (**b**) The harvested culture was separated into the culture solution and cyanobacterial cells via centrifugation; water was directly added to the cyanobacteria cells and the mixture was allowed to stand in the dark at 30°C. (**c**) After lysis, phycocyanin (PC) aqueous solution was obtained via centrifugation. To remove impurities present in the aqueous PC solution, 0.5% (w/v) activated carbon was added to the PC aqueous solution and stirred at room temperature for 15 min. Thereafter, activated carbon was precipitated via centrifugation to recover the phycocyanin aqueous solution. (**d**) PC powders were obtained after freeze-drying the PC solution.

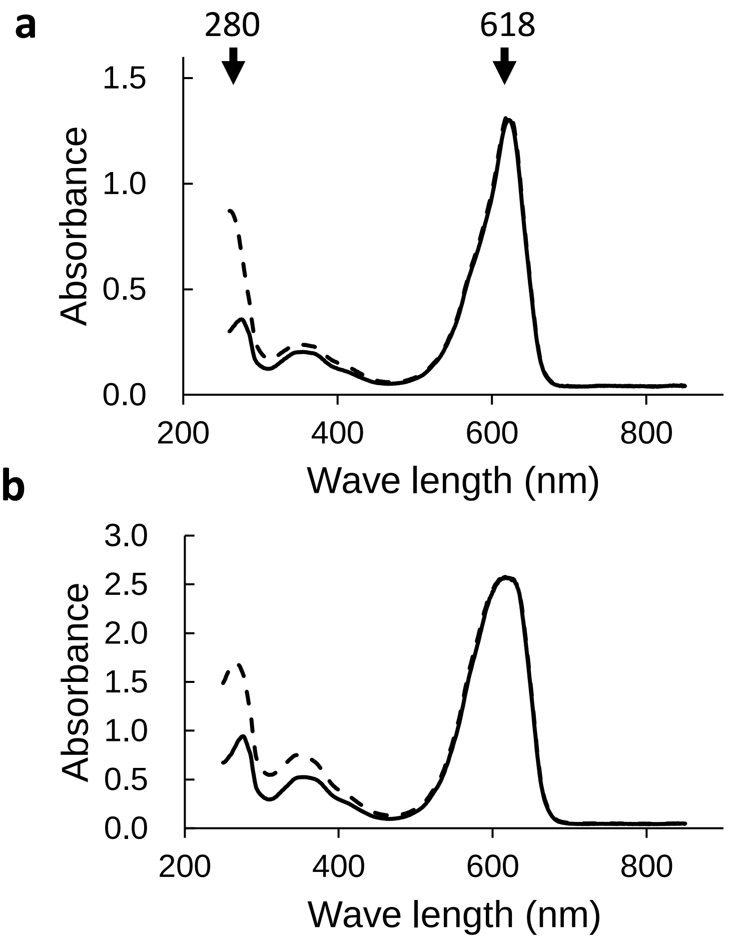

Figure S3 Absorption spectrum for the phycocyanin fraction. Absorption spectra of phycocyanin fractions treated without or with activated carbon are shown as broken or solid lines, respectively. (**a**) The purity values of ABRG5-3 (OD618/OD280) are 1.76 or 3.10 without or with activated carbon treatment, respectively. (**b**) The purity values of SK1-2-1 were 1.79 or 2.14 without or with activated carbon treatment, respectively.

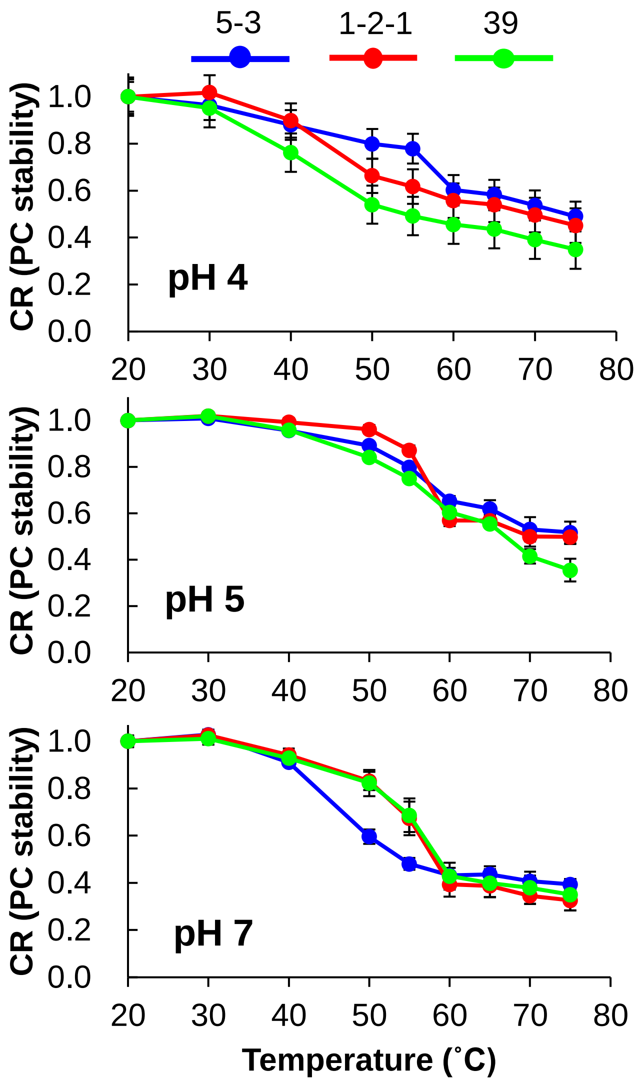

Figure S4 Stability of phycocyanin depends on pH under heat. The phycocyanin solutions of pH 4, 5, and 7 were incubated at temperatures increasing in steps of 9 degrees each time from 20 to 75°C for 30 min. The OD618 and OD652 values were measured for CR (phycocyanin stability). The mean of the values obtained from three independent experiments is shown together with standard errors.

CpcA

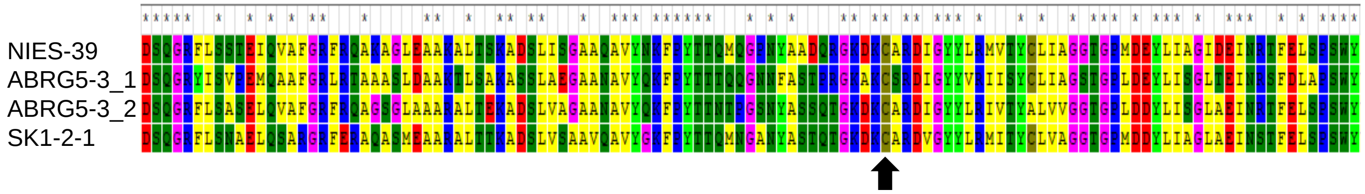

CpcB

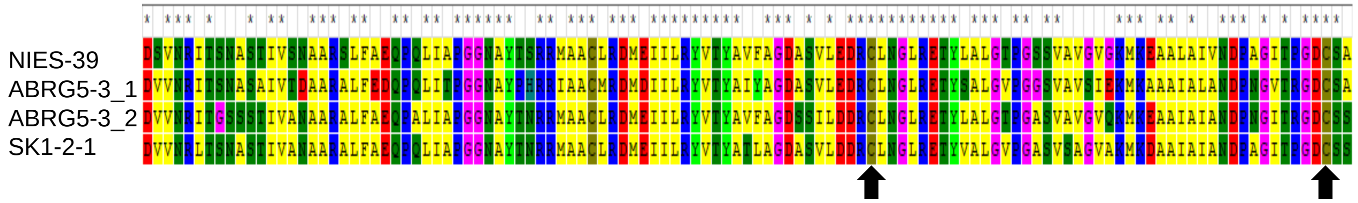

Figure S5 Alignment of the amino acids sequence of phycocyanin subunits. (Upper) CpcA (α-subunit) showing a cysteine residue (arrow) involved in binding to phycocyanobilin in NIES-39, ABRG5-3, and SK1-2-1. Asterisks indicate identical residues. (Lower) Homology of the CpcB (β-subunit) among NIES-39, ABRG5-3, and SK1-2-1.
