# Supplementary material for: Development of a method for phycocyanin recovery from filamentous cyanobacteria and evaluation of its stability and antioxidant capacity
Source: BMC Biotechnol. 2021 Jun 16;21:40. doi: 10.1186/s12896-021-00692-9 (PMC8210370; doi:10.1186/s12896-021-00692-9)
Supplement: Supplementary file 2 — Additional file 2: Table S1. Extraction, yield, and purity of phycocyanin from cyanobacteria. [file 12896_2021_692_MOESM2_ESM.pdf]

Additional file 2: Additional table

BMC Biotechnology

Development of a method for phycocyanin recovery from filamentous cyanobacteria and evaluation of its stability and antioxidant capacity

Jinichi Aoki[1,2], Daisaku Sasaki[3], Munehiko Asayama[1,2*]

[*]Correspondence: munehiko.asayama.777@vc.ibaraki.ac.jp

[1]College of Agriculture, Ibaraki University, 3-21-1 Ami, Ibaraki 300-0393, Japan
[2]United Graduate School of Agricultural Science, Tokyo University of Agriculture and Technology, 3-5-8 Saiwai-cho Fuchu-shi, Tokyo 183-8509, Japan
[3]BioX Chemical Industries Co. Ltd., 2-20-11 Inokuchidai, Nishi-ku, Hiroshima 733-0844, Japan

Table S1 Extraction, yield, and purity of phycocyanin from cyanobacteria

Cyanobacteria	Medium	Extraction		Cells (W/D)	PC (μg mL^{-1})	Yield (%)	Purity (A620/A280)	References
		Method	Solvent					
Pseudanabaena sp. ABRG5-3	BG11	Water addition	Water	Wet	119	30.4	3.10	This study
Limnothrix sp. SK1-2-1	BG11	Water addition	Water	Wet	133	28.9	2.14	This study
Spirulina platensis (NIES-39)	BG11	Water addition	Water	Wet	39	7.8	1.76	This study
Spirulina platensis (NIES-39)	SOT	Water addition	Water	Wet	164	21.1	1.76	This study
Spirulina platensis (CCC540)	Z-medium	Freezing and thawing	Acetate buffer, sodium chloride, sodium azide	Wet	77	-	0.75	Kumar et al., 2014
Spirulina platensis (CCC540)	Z-medium	Ammonium sulphate precipitation	Acetate buffer, sodium chloride, sodium azide	Wet	124	-	1.50	Kumar et al., 2014
Spirulina platensis (CCC540)	Z-medium	Dialysis	Acetate buffer, sodium chloride, sodium azide	Wet	601	-	2.93	Kumar et al., 2014
Spirulina platensis (CCC540)	Z-medium	DEAE column chromatography	Acetate buffer, sodium chloride, sodium azide	Wet	413	-	4.58	Kumar et al., 2014
Spirulina platensis	-	Conventional separation	Phosphate buffer	Dry	-	7.2	0.88	Seo et al., 2013
Spirulina platensis	-	Hexane extraction with high pressure	Phosphate buffer, hexane	Dry	-	10.2	0.91	Seo et al., 2013
Spirulina platensis	-	Hexane extraction	Phosphate buffer, hexane	Dry	-	8.4	0.88	Seo et al., 2013
Cylindrospermopsis raciborskii	Modified-BG11	Freezing and thawing	Phosphate buffer	Wet	2	-	-	Horváth et al., 2013
Cylindrospermopsis raciborskii	Modified-BG11	Grinding	Phosphate buffer	Wet	3	-	-	Horváth et al., 2013
Cylindrospermopsis raciborskii	Modified-BG11	Sonication	Phosphate buffer	Wet	3			Horváth et al., 2013
Cylindrospermopsis raciborskii	Modified-BG11	Homogenization with polytron	Phosphate buffer	Wet	1	-	-	Horváth et al., 2013
